# Validity and reliability of the Beck Depression Inventory (BDI-II) in general and hospital population of Dominican Republic

**DOI:** 10.1371/journal.pone.0199750

**Published:** 2018-06-29

**Authors:** Zoilo Emilio García-Batista, Kiero Guerra-Peña, Antonio Cano-Vindel, Solmary Xiomara Herrera-Martínez, Leonardo Adrián Medrano

**Affiliations:** 1 Pontificia Universidad Católica Madre y Maestra, Santiago de los Caballeros, Dominican Republic; 2 Universidad Complutense de Madrid, Madrid, Spain; 3 Universidad Arturo Michelena, Valencia, Venezuela; 4 Universidad Siglo 21, Córdoba, Argentina; 5 Universidad Nacional d Córdoba, Córdoba, Argentina; Hong Kong Polytechnic University, HONG KONG

## Abstract

The Beck Depression Inventory-II (BDI-II) is currently one of the most widely used measures in both research and clinical practice for assessing depression. Although the psychometric properties of the scale have been well established through many studies worldwide, so far there is no study examining the validity and reliability of BDI-II in Republic Dominican. The purpose of the present study was twofold: (a) to examine the latent structure of BDI-II by testing several competing models proposed in the literature; and (b) to provide evidence of validity and reliability of the BDI-II in Republic Dominican. Confirmatory factor analysis indicated that a bifactor model with a general depression factor and three specific factors consisting of cognitive, affective and somatic showed the best fit to the data. Internal reliability was moderate to high for all subscales and for the total scale. Scores on BDI-II discriminated between clinical and general population, supporting for external validity. Practical implications are discussed and suggestions for further research are also made.

## Introduction

Depression is a common mood disorder that affects individual’functioning individual ‘functioning across different domains. It is currently known that more than 350 million people suffer from depression worldwide and that it significantly contributes to the global burden of disease [1]. Depression stands out not only for its high prevalence, but also due to the probability of associated relapse and recurrence. Another setback is the high financial cost that it entails, which translates into low productivity, workplace absenteeism, outpatient care, hospitalizations and pharmacological treatments [2].

According to the World Health Organization [3] depression is the leading cause of years lived with disability (YLD), and the most prevalent disorder among serious psychiatric disorders in primary care setting. This disorder is characterized by changes in sleep, appetite and psychomotricity, decreased concentration and decision-making ability, loss of self-confidence, feelings of inferiority or worthlessness and guilt, as well as despair and recurrent thoughts of death with ideation, planning and/or suicidal acts.

So far, the Beck Depression Inventory-II (BDI-II) has become one of the most widely used measures to assess depressive symptoms and their severity in adolescents and adults [4]. The BDI-II [5] is a 21-item self-report measure that taps major depression symptoms according to diagnostic criteria listed in the Diagnostic and Statistical Manual for Mental Disorders [6]. Items are summed to create a total score, with higher scores indicating higher levels of depression. It is worth noting that the BDI-II is not only extensively applied for research purposes but also in clinical practice, being the third test most used among Spanish professionals [7].

Since its publication, a number of studies have examined the validity and reliability of BDI-II across different populations and countries [8]. Results have consistently shown good internal consistency and test-retest reliability of the BDI-II incommunity [9,10,11] adolescent and adult clinical outpatients [12] as well as in adult clinical inpatients [13]. Criterion-based validity have also shown acceptable sensitivity and specificity of the BDI-II for detecting depression, supporting its clinical utility as an aid measure for diagnostic purposes [2,14,15]. In contrast, findings concerning BDI-II factor structure have been somewhat inconsistent. Particularly, while Beck et al. [16] two-factor correlated model composed of a cognitive-affective and a somatic factors has been supported in many studies [17,18,19]; there are others studies which identified a single factor [11,20], two alternative factors consisting of somatic-affective and cognitive [21,22], three factors corresponding to cognitive, somatic and affective [23,24,25], and an alternative three-factor model including negative attitude, difficulty and somatic [23–29]. Less frequently, four [30] and fivefactors [31] have also been reported.

Additionally, more sophisticated analysis into the BDI-II factor structure including hierarchical and bifactor models have been tested. Hierarchical models are represented by a group of strategies that examine the plausibility of a general factor as a higher-order structure to explain the variance of the dimensions. Bifactor models, in contrast, allow to examine a non-hierarchical general factor independently of the specific factors and to simultaneously test the extent to which the common variance between items are explained by the orthogonal general factor and by the specific factors that are tested [32]. By doing so, bifactor models represent a useful strategy to examine if a construct of interest can be viewed primarily as unidimensional or multidimensional and, subsequently, the way in which scores should be computed.

Results from hierarchical and bifactor BDI-II models supported both models. For example, Byrne et al. [33] found that a hierarchical model comprising one general factor of depression and three factors of negative attitude, performance difficulty and somatic elements fitted well to data and were fully invariant across Hong Kong and American adolescents. Subica et al. [13] compared a unidimensional model, three alternative two-factor models and three bifactor models including an independent general depression factor and specific factors. They found that none of the two-factor models have acceptable fit and, in contrast, all the corresponding bifactor models showed good fit indices, concluding that only BDI-II total score should be used to measure the severity of depression. Similarly, McElroy et al. [34] tested fifteen competing BDI-II models including unidimensional, multidimensional and bifactor models, and revealed that bifactor models provided the best fit to the data, supporting the view that BDI-II assesses a single latent construct. Finally,Vanheule et al. [35] did not find confirmatory evidence for bifactor models but, instead, they found that a three-factor model consisting of affective, cognitive and somatic factors provided better fit to data in clinical and non-clinical samples.

### Current study

In summary, although factorial data suggests that bifactor models outperform multidimensional models–regardless of the number of specific factors–findings are not conclusive [36,37,38]. Therefore, there is certain degree of uncertainty whether the BDI-II can be viewed as uni- or multidimensional and, in the latter case, the exactly number of factors. Thus, further research is needed into the latent structure of BDI-II. Addressing this issue may have not only practical implications (i.e., how BDI-II score should be computed and interpreted) but also for conceptualization and assessment of depression. Moreover, despite that cultural convergence is being accelerated due to increased globalization [39] and that major depression has been reported worldwide [40] there are considerable cross-cultural differences in the symptomatology of depression [41]. To the extent that depression’ symptoms and inner experience may differ across cultural backgrounds [42], findings cannot be generalized. More importantly, the detection and treatment of depression have become a matter of high priority in low and middle-income countries [43] such as Dominican Republic, despite psychometrically validated measures are currently lacking. Therefore, the purpose of the present study was twofold. First, to determine the most appropriate BDI-II factor structure by examining several competing factor models that have been reported in previous studies. Secondly, to examine the validity and reliability of BDI-II in Dominican Republic.

## Method

"This research had the revision and approval of the National Council of Bioethics in Health/ Consejo Nacional de Bioética en Salud (CONABIOS) of the Dominican Republic. The protocol registration number in CONABIOS was 028–2014."

### Participants

One thousand and forty individuals (54.9% women and 45.1% men) from Dominican Republic participated in the study. The mean age was 27.07 (SD = 11.18). Participants were selecting by convenience from general population (N = 797) and hospital population (N = 243). Within the hospital sample, 76.5% came in for routine checkups, 15.3% sought help for cardiac and hypertension conditions and 8.2% went to the psychiatric service.

## Measure

Depression Inventory–II (BDI–II) [5]. The Self-report study based on the symptoms described by the Diagnostic and Statistical Manual of Mental Disorders (DSM-IV) [6], which makes measuring depressive severity possible. This version of the inventory consists of 21 items, in which four response options are presented on a scale of 0 to 3. For example, to measure pessimism (item 2) the response options used range from “I am not particularly discouraged about the future” (score of 0) to “the future is hopeless and things cannot improve” (score of 3). In this study we are using the Spanish version of Beck Depression Inventory-II [44], which has an excellent reliability coefficient of .92. Its content validity is ensured because most of its items are equivalent to the DSM-IV criteria for depression. Its construct validity has also been tested successfully by comparing scores with other measures for depression.

### Procedure and data analysis

While Dominicans’ native language is the same that the language BDI-II version used in this study (i.e., Spanish), there are linguistic characteristics that may vary substantially. Thus, even using the exactly same words the interpretation and meaning may be quite different [45]. Therefore, a pilot study was first conducted to ensure that participants correctly understood the content of BDI-II items. Fifteen people were asked to complete the scale and write down items that were unclear or incomprehensible, as well as any other aspect of the scale that may deem relevant. Once the activity was completed, a focus group was used to enable individuals to share their appreciations concerning items, response format, instructions, and to check for discrepancies in the interpretation or meanings. There was neither difficulty in understanding nor negative commentaries about the scale content. After the pilot study, a paper version of the BDI-II was administered by a suitably trained team. All participants agreed to participate voluntarily and provided written consent prior to complete the inventory and after information about purposes of the study were provided. Preliminary analysis using SPSS v20 was carried out to examine outliers, missing values and to test assumptions of univariate and multivariate normality. Next, internal structure of the BDI-II was assessed using confirmatory factor analysis (CFA) through AMOS v20 [46]. Since Mardia’s kurtosis multivariate coefficient was 338.70 –thus indicating a significant deviation from multivariate normality according to benchmarks [47]–the Asymptotic Distribution-Free method was used for model estimation. To examine model fit,the chi-square value (χ^2^), the comparative fit index (CFI), the goodness-of-fit index (GFI), the Tucker-Lewis index (TLI) and the Root Mean Square Error of Approximation (RMSEA). The cut-off points of values greater than .95 reported by Hu and Bentler [48] and Joreskog and Sorbom’s [49] were used for the CFI and GFI indices in order to consider an optimal fit, and greater than .90 for an acceptable fit. On the other hand, values lower than .06 for the RMSEA are considered optimal and lower than .08 are considered acceptable. As an additional criterion, the χ² value was divided by the degrees of freedom (χ²/df), with the aim of obtaining values lower than 3 in order to consider the model a good fit [50,51]. In order to compare the fit of the models, the Akaike Information Criterion (AIC) was also considered. According to this index, those models that present values lower than AIC provide a better fit. Also, when verifying the fit of a bifactor model, it makes sense to consider additional indices, mainly the hierarchical omega (*ω*_*H*_), the percentage of explained common variance (ECV) and the percentage of uncontaminated correlations (PUC).

In addition, the internal consistency was evaluated using Cronbach's α statistic, and validity evidence was provided by comparing the BDI-II scores of the general population and the hospital population; to do so, successive Student's t tests were carried out for independent samples applying a Holm–Bonferroni adjustment to control for Type 1 error. In this case, a stepwise procedure is used where each *p-*value is compared with α/(*n—i* + 1) for rejection. The comparison continues in a sequential increasing order (from *i-*1 and proceeding in order) until the first nonrejection. This method has demonstrated to be statistically more powerful for controlling Type 1 error compared to Bonferroni adjustment [52]. In order to assess the effect size of these differences, Cohen's d was calculated, with values around .30 considered as small effects, values around .50 as medium effects and values greater than .80 as large effects [53].

## Results

### Model comparisons

**Fig 1:**
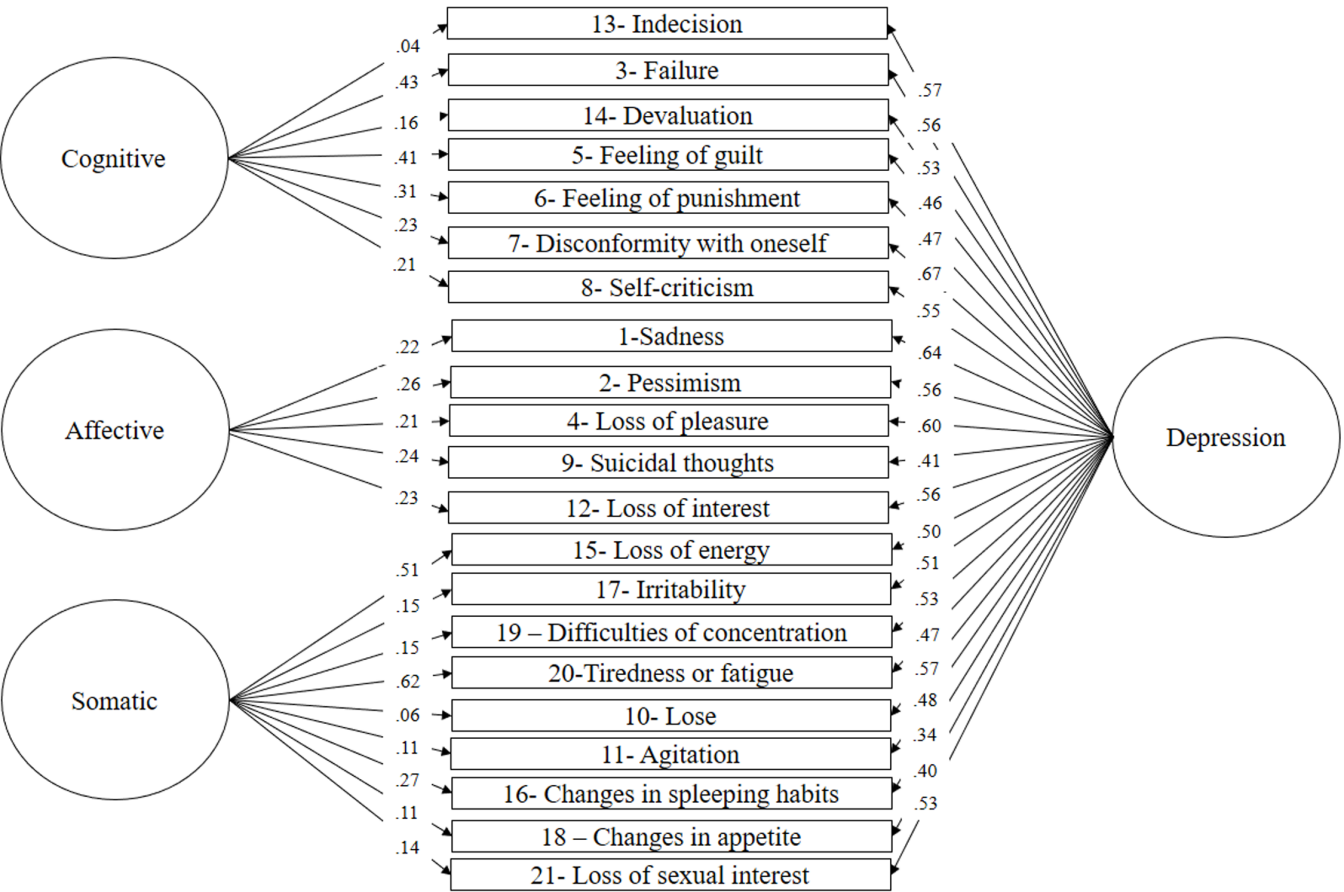
Bifactor model with a general depression factor and three specific factors consisting of cognitive, affective, and somatic factors.

Based on previous BDI-II research findings, several competing models were tested including one, two, three-factor models and bifactor models. In particular, *Model 1* assumes depression as a unitary construct and, therefore, all BDI-II items were allowed to load into a single factor (“Depression”) [20]; *Model 2* tested a two-factor model represented by “cognitive-affective” and “somatic” factors [45]; *Model 3* tested the original two-factor model identified by Beck et al. [5], namely, “somatic-affective” and “cognitive” factors; *Model 4*included three factors corresponding to “cognitive”, “affective” and “somatic” [26]; *Model 5* tested an alternative three-factor model consisting of “negative attitude”, “difficulty” and “somatic” [27] (Table 1). Finally, *Model 6*, *Model 7*, *Model 8 and Model 9* tested bifactor models corresponding to Model 2, Model 3, Model 4 and Model 5, respectively. In these models, an orthogonal general factor called “depression” was tested along with the specific proposed factors. Results are summarized in Table 2. In general, neither the unidimensional model nor the one, two and three factor models reached acceptable fit indices. In contrast, all the corresponding bifactor models fitted well to the data. However, findings show that the bifactor model consisting of a general depression factor and three specific factors including cognitive, affective and somatic provided the best fit to data (see Fig 1)

**Table 1:** Models of Beck Depression Inventory-II (BDI-II).

	Model 1	Model 2		Model 3		Model 4			Model 5		
Items	D	C-A	S	S-A	C	C	A	S	NA	D	S
1	X	X			X		X		X		
2	X	X			X		X		X		
3	X	X			X	X			X		
4	X		X	X			X			X	
5	X	X			X	X			X		
6	X	X			X	X			X		
7	X	X			X	X			X		
8	X	X			X	X			X		
9	X	X			X		X		X		
10	X		X	X				X			X
11	X		X	X				X			X
12	X		X	X			X			X	
13	X	X		X		X				X	
14	X	X			X	X			X		
15	X		X	X				X		X	
16	X		X	X				X			X
17	X		X	X				X		X	
18	X		X	X				X			X
19	X		X	X				X		X	
20	X		X	X				X		X	
21	X		X	X				X			X

*Note*: D: Depression; C-A: Cognitive-affective; S: Somatic; S-A: Somatic-Affective; C: Cognitive; NA: Negative Attitude; D: Difficulty

**Table 2:** Fit indices for all the specified models of Beck Depression Inventory-II (BDI-II).

	χ^2^	df	χ^2^/df	CFI	GFI	TLI	RMSEA (90% CI)	Akaike Information Criterion (AIC)
*Model 1*: Unidimensional model	968.61***	189	5.12	.87	.91	.85	.063 (.059-.067)	1052.61
*Model 2*: Cognitive-affective and somatic	823.48***	188	4.38	.89	.93	.88	.057 (.053-.061)	909.48
*Model 3*: Somatic-affective and cognitive	795.97***	188	4.23	.90	.93	.88	.056 (.052-.060)	881.97
*Model 4*: Cognitive, affective and somatic	772.84***	186	4.15	.90	.93	.88	.055 (.051-.059)	862.84
*Model 5*: Negative attitude, difficulty and somatic	792.94***	186	4.26	.90	.93	.88	.056 (.052-.060)	882.94
*Model 6*: Bifactor model (with cognitive-affective and somatic as specific factors)	560.59***	168	3.33	.93	.95	.92	.047 (.043–0.52)	686.59
Model 7: Bifactor model (with cognitive and somatic-affective as specific factors)	550.04***	168	3.27	.94	.95	.92	.047 (.042-.051)	676.04
Model 8: Bifactor model (with cognitive, somatic and affective as specific factors)	541.57***	168	3.22	.94	.95	.92	.046 (.042-.051)	667.57
Model 9: Bifactor model (with negative attitude, difficulty and somatic as specific factors)	561.28***	168	3.34	.93	.95	.92	.047 (.043-.052)	687.29

*Note*: df: degree of freedom; CFI: comparative fit index; GFI: goodness of fit index; TLI: Tucker-Lewis index; RMSEA: root mean square error of approximation.

***p<.001

Complementary indices were calculated to evaluate the fit of the bifactor model, including *ω*_*H*_, the ECV and PUC. In this study a *ω*_*H*_of .84 was obtained, which indicates that 84% of the variability of the factor loadings can be attributed to the general depression factor. The .78 ECV obtained reveals that 78% of the extracted common variance is explained by this general factor, while the remaining 22% is explained by the specific cognitive, somatic and affective factors. Finally, the PUC value indicates that 68% of the correlations are influenced by the general factor. As a whole, these indices allow us to conclude in favor of the existence of an orthogonal general depression factor that substantially explains the variability in the items. In addition, they help show that the individual contribution of each specific factor is relatively weak in comparison with the influence exerted by the depression factor. This is perhaps better expressed in terms of average factorial loads, where a greater influence on general factor items (*λ*_average_ = .52) is observed compared to those observed for the specific cognitive (*λ*_average_ = .26), affective (*λ*_average_ = .23) and somatic (*λ*_average_ = .24) factors.

### Internal consistency

The internal consistency of each factor and the general scale was assessed using Cronbach's α coefficient. The corrected item-total correlation was also calculated for the items of each factor. The results obtained were: α = .78 for the Cognitive dimension (corrected item-total correlations between .45 and .62); α = .77 for the Somatic dimension (corrected item-total correlations between .42 and .56); and α = .70 for the Affective dimension (corrected item-total correlations between .37 and .55). The total scale was the only one that presented values greater than .80 (α = .89; corrected item-total correlations between .37 and .62).

### Known-group validity

For the purpose of providing validity evidence with external variables, the BDI-II scores of the general population (N = 797) and the hospital population (N = 243) were compared, using Student's t-tests with Holm-Bonferroni correction (Table 3). As expected, statistically significant differences were observed with higher averages in the hospital sample. These differences are amplified when the hospital sample is sub-divided and only the psychiatric consultation participants (N = 86) are considered. In effect, higher averages are observed in the general BDI-II score (average = 16.91; standard deviation = 11.62; *t* (881) = 7.49; *p*<0.01), and in the cognitive dimensions (average = 5.02; standard deviation = 4.31; *t* (881) = 6.33; *p*<0.01), somatic (average = 8.24; standard deviation = 5.35; *t* (881) = 5.87; *p*<0.01), and affective (average = 3.65; standard deviation = 3.15; *t* (881) = 8.17; *p*<0.01), with a greater effect size on the Cohen's d values (*d* general = .80; *d* cognitive = .64; *d* somatic = .71; *d* affective = .82).

**Table 3:** Comparison of BDI-II scores between general population (N = 797) and hospital population (N = 243).

	General		Hospital			
	M	SD	M	SD	*T*	Cohen’s *d*
Cognitive	2.63	2.94	3.83	4.14	-5.01**	0.33
Somatic	4.93	3.74	8.00	5.27	-10.09**	0.64
Affective	1.53	1.85	2.84	2.93	-8.30**	0.53
Total	9.09	7.40	14.66	10.99	-9.07**	0.59

** *p*<.01 (with Holm–Bonferroni correction)

## Discussion

Depression represents the fourth leading cause of disability worldwide [3] with the higher prevalence in low and middle-income countries [54]. In Dominican Republic the scientific research on depression is absent [55] which may negatively impact the development of cultural sensitive evidence-based interventions. From a practical standpoint, the lack of assessment tools for depression may not make available protocols for early identification of depression symptoms at primary care units. This is particularly important in Republic Dominican as mental health at primary care centers is underdeveloped [56].

The present study sought to examine the dimensionality and reliability of the BDI-II in Republic Dominican. Several factor structure models, including one-factor, two-factor, three-factor and bifactor models were tested with the purport to determine the optimal factor structure. Results showed that a bifactor model with a general depression factor and three specific factors consisting of cognitive, affective, and somatic factors provided the best fit to data. This is in line with different studies that supported a bifactor latent structure to the BDI-II [13,36,37,38]. In practice, this finding implies that BDI-II items can be summed to form an overall score, with higher total scores indicating greater level of depression severity [32]. Moreover, despite most of the items variances were accounted by the general depression factor, the three specific factors (cognitive, affective, and somatic) explained a non-redundant amount of variance. Thus, in contrast to different authors who advocate the use of BDI-II total scores and questioned the validity of subscales [13,34], the present findings support the use of the BDI-II total score along with scores corresponding to each subscale, in agreement with Beck et al. [5] original scoring instructions. Furthermore, since research indicates that depression symptoms response differentially to treatment [57] the use of BDI-II global score alone as a measure to detect changes in response to treatment may obscure the impact of interventions. In conclusion, for both statistical and clinical reasons it seems more appropriate to use BDI-II total and factor scores.

Additionally, the present study supported the validity of the affective factor as a separate dimension from cognitive and somatic domains. This finding differs from common findings indicating that the affective factor should be subsumed by the cognitive [17,18,58] or the somatic factor [5,10,59,60]. According to Steer et al. [61], affective symptoms may shift from one dimension to another depending on background and composition of samples being studied. As such, it would be valuable to test the invariance measurement of the BDI-II factor structure found in this study across different samples in order to examine the robustness of the affective component as a single and differentiated domain of depression.

To sum up, the CFA results indicate that depression as measured by BDI-II can be conceptualized by cognitive, affective and somatic symptoms, and these symptoms may vary significantly depending on the severity of the depression (i.e., the depression general factor). Subsequent reliability analysis of the BDI-II total score and subscale scores showed acceptable to high internal consistency, with alpha coefficients ranging from .70 to .89. As expected, t-test analysis revealed that BDI-II scores discriminated between individuals from hospital and general population. Collectively, these results support the use of BDI-II in Republic Dominican for assessing depression severity.

Notwithstanding the implications aforementioned, the current study has a number of limitations that should be mentioned. First, the sample study was selected by convenience being primarily compounded by individuals stem from general population. Therefore, findings cannot be generalized and further replication in both representative samples from general population and clinical samples are needed. Second, we focused in the analysis of the latent structure of BDI-II, which is just one element of construct validity among several others [62]. Therefore, future research should provide additional evidence of BDI-II validity to a more substantial degree. In particular, it would be worthwhile to further examine the capacity of BDI-II scores to discriminate between depressed and non-depressed subjects. Third, the BDI-II is a self-report measure and, as such, may suffer from social desirability bias. Indeed, Hunt et al [63] demonstrated that subjects who administered a manipulated version of BDI-II in which the purpose was disguised and the content was padded with items that not tap depression symptoms, scored significantly higher than subjects who completed the original scale. Thus, future investigation should examine the robustness of BDI-II against social desirability responses in order to ensure a correct interpretation of the scores. Fourth, it has been suggested that bifactor models are more robust to model misspecification (e.g., substantive cross-loadings) than multidimensional or hierarchical CFA models [64] which may result in bias in favor of bifactor models and, consequently, to explain why such measurement models outperform conventional CFA models. Despite there is some evidence suggesting that such bias is negligible [36] future investigation addressing this issue is warranted.

Despite all limitations, we note that this is the first study to demonstrate the construct validity and reliability of the BDI-II in Dominican Republic. The lack of psychometrically well-established measures for assessing depression in community hinder the early detection of symptoms, the evaluation of the effectiveness of interventions and the development of research programs aimed to identify risk factors associated to depression in Dominican population. Hopefully, this study will help to change this situation.

## Supporting information

S1 File Dataset. BDI dataset in SPSS format.
(SAV)
